# Aspirin Effect on *Staphylococcus aureus*—Platelet Interactions During Infectious Endocarditis

**DOI:** 10.3389/fmed.2019.00217

**Published:** 2019-10-15

**Authors:** Nadji Hannachi, Gilbert Habib, Laurence Camoin-Jau

**Affiliations:** ^1^Aix Marseille Univ, IRD, APHM, MEPHI, IHU Méditerranée Infection, Marseille, France; ^2^Département de Cardiologie, Hôpital de la Timone, AP-HM, Marseille, France; ^3^Laboratoire d'Hématologie, Hôpital de la Timone, APHM, Marseille, France

**Keywords:** aspirin, platelets, *Staphylococcus aureus*, infectious endocarditis, embolic events

## Abstract

Infectious endocarditis (IE) is a rare disease associated with high mortality and morbidity rate. The platelet–bacterial interaction presents the cornerstone of the development of endocardial vegetation. The epidemiology of IE has undergone profound changes between the last and the new decade, with *Staphylococcus aureus* becoming the main incriminated species. Despite improvements in antibiotic and surgical therapies, embolic disorders remain highly associated with IE that can be fatal. Antiplatelet drugs have been widely proposed to overcome embolic events associated with IE. This proposal has been supported by numerous *in vitro*, experimental, and clinical studies. However, other studies have yielded conflicting results. In this review, we focus on the effect of aspirin on the genesis of *S. aureus* endocarditic vegetation, as well as on the management of embolic and hemorrhagic events related to it, starting by its influence on the platelet–bacteria interaction.

## Introduction

Infectious endocarditis (IE) is a serious disease related to high mortality and morbidity. It is an infection of the endocardium and heart valves or prosthetic valve implant that can be caused by a multitude of bacterial and mycotic species (1). IE is characterized by the development of vegetation, which is mainly formed by platelets, fibrin, and the microbial agent. Despite improvement in antimicrobial treatment and surgical intervention, the evolution of the disease can be complicated by many events including embolism, present in a fifth to a third of patients (2, 3). Embolic events remain a major complication and are one of the leading causes of death for which no pharmacological treatment is dedicated yet (4). The evolution of hospital practices and the use of antibiotics have contributed to the change in the microbiological profile of IE. *Staphylococcus aureus* becomes now the predominant causative agent in the developmental world (5–7).

Antiplatelet agents, mainly aspirin, have been widely proposed for the treatment or prevention of IE, primarily the prevention of related embolic events. Some studies have concluded to the effectiveness of aspirin in this indication. However, other studies, both experimental and clinical, have demonstrated the opposite. In this review, we describe the effects of aspirin on the interaction between platelets and *S. aureus* during endocardial vegetation growth provided by *in vitro* and experimental investigations, as well as its clinical efficacy in the management of embolic and hemorrhagic events during native and prosthetic valve IE. Cardiac implanted electronic device related endocarditis is not covered in this review.

## Epidemiology of *S. aureus* Endocarditis

IE represents a relatively rare pathology associated with high mortality rate reaching 25%, even with adequate therapy (5). Its global impact ranges from 1.5 to 11.6 cases per 100,000 person-years (8). The microbiological profile of IE has undergone profound changes, notably in terms of the importance of causal agents. With the decrease in the occurrence of acute articular rheumatism caused by *Streptococcus*, mainly in the developed countries, and the emergence of new risk factors (intravenous drug addiction, prosthetic valve, degenerative valve sclerosis associated with aging of the population, performing invasive acts at risk of bacteremia), *S. aureus* becomes the leader agent responsible for IE (9, 10), the latter being contracted in 10–25% of *S. aureus* bacteremia (11, 12).

Results reported in the International Collaboration on Endocarditis–Prospective Cohort Study, in 4,049 cases of IE, showed that most cases were attributable to *S. aureus* (30.1%), with 17.3% *viridans streptococci* and 9.4% negative coagulase *staphylococci* (2). In addition, in a recent prospective cohort of the European infective endocarditis registry (EuroEndo) conducted on 3,116 patients mainly from Europe, *S. aureus* was involved in 44.1% of cases and formed a risk factor for embolic events, followed by *Entrococci* (15.8%), *viridans streptococci* (12.3%), and *Streptococcus galloliticus* (6.6%) (3). Nosocomial cause is continuously increasing with a rate of nearly 60% due to *Staphylococcus* genus (13).

According to three population studies conducted in several French regions totaling 11 million inhabitants and including prospectively all the patients treated for an IE that were carried out in 1991, 1999, and 2008, the incidence remained stable over time, with an average of 35 cases per million inhabitants. According to the same studies, the evolution of the ratio of male to female increased from 1.91 in 1991 to 2.94 in 2008, suggesting that other gender-related risk factors are still unmatched to date (14–16).

The production of reliable epidemiological data on IE is a critical step due to the presence of several limitations. Among them are the heterogeneity between the data of the different continents due to the differences on hospital practices and the evolution of the IE, which is closely linked to it. In addition, population studies are subject to sampling bias with a risk that the population studied may not truly represent the general population. Studies conducted in hospitals can also have a reference bias, with sicker patients being referred to specialized centers. Thus, these results may not apply to community hospitals (2, 17). Data from the European infective endocarditis registry will probably provide the first piece of reliable epidemiological evidence of IE at the continental level (3).

## Pathophysiology

Classically, the generation of IE begins with an endothelial lesion, to which platelets and fibrin adhere. During an episode of bacteremia, the microbial agent finds this site favorable for nesting, which results in the formation of vegetation located in the inner wall of the heart or on a prosthetic valve. In the next section, we will focus only on the physiopathology of IE caused by *S. aureus*.

*Staphylococcus aureus* is a versatile bacterium with a varied arsenal of components, including toxins, enzymes, and surface molecules that act either alone or in concert, making it a remarkable species whose virulence can range from simple colonization to sever systemic infections (18, 19). *S. aureus* bacteremia is related to invasive procedures, such as catheter location, administration of intravascular drugs, or any form of surgery (20, 21). IE, whether due to *S. aureus* or other bacterial species, is related to the accumulation of low levels of bacteria in the bloodstream rather than a massive bolus introduction (22).

### Vegetation Growth

Vegetation growth is a complex phenomenon involving mainly an interaction between platelets and bacteria*. S. aureus* expresses several membrane motifs that interact with platelets (Table 1). Among them, protein A, an immunogenic surface protein, can bind platelet FcγRII via an immunoglobulin G antibody or GPIbα via von Willebrand factor (25, 26). The staphylococcal accessory regulator (Sar) P protein can directly bind to GPIbα through its sialic acids (24). Clomping factor A is also a membrane protein of *S. aureus*, which is a member of microbial surface components recognizing adhesive matrix molecules family. It binds to platelets via fibrinogen and plays a key role in the development of vegetation (24, 27). Clomping factor B and serine-aspartate repeat protein E (SdrE) are also involved in platelet aggregation (28, 29). *S. aureus* produces two homologs of fibronectin binding proteins A and B, which mainly bind fibronectin and fibrinogen (30). They also bind platelet FcγRIIa via specific immunoglobulin G antibody (32). *S. aureus* expresses in its cell wall a protein called iron-responsive surface determinant, which can bind to the heme of hemoglobin and thus allows its internalization by the bacterium and its use as a source of iron. Iron-responsive surface determinant B binds directly GPIIb-IIIa (31). Furthermore, certain toxins are involved in the process of adhesion. Thus, the α toxin, in addition to the induction of a membrane alteration, induces activation and platelet aggregation (23). In addition, staphylococcal superantigen-like, another staphylococcal toxin, can adhere to platelets on GPIbα and GPVI (24).

**Table 1:** *S. aureus*–platelet interactions.

**Nature**	***S. aureus* motifs**	**Plasma proteins**	**Platelet receptors**
Toxins	Alpha toxin	–	Lipid bilayer membrane
	SSL5	–	GPIbα
		–	GPVI
Membrane Proteins	Protein A	IgG	FcγRII
		vWF	GPIbα
	Sar P	–	GPIbα
	Clf A and B	Fibrinogen	GP IIbIIIa
	SdrE	?	?
	FnBP A and B	Fibrinogen	GP IIbIIIa
		Fibronectin	
		IgG	FcγRII
	IsdB	–	GP IIbIIIa
	?	C1q	gC1q-R
	?	C3b	P selectin
SERAM	Eap	–	Glycosaminoglycan

*Summary table of the main ligand receptor pairs on both sides of the platelets and S. aureus, requiring or not intermediate plasma proteins. SERAM, secreted expanded repertoire adhesive molecules; SSL5, staphylococcal superantigen-like 5; Sar P, staphylococcal accessory regulator protein; Clf, clumping factor; Sdr E, serine-aspartate repeat protein E; FnBP, fibronectin binding protein; Isd B, iron-responsive surface determinant B; Eap, extracellular adherence protein. vWF, Von Willebrand Factor;?, unknown (23–32)*.

Moreover, *S. aureus* acts on coagulation step through the secretion of two coagulases: staphylo-coagulase and von Willebrand factor binding protein (33, 34). Staphylococcal coagulases form a complex with prothrombin called staphylothrombin. The latter transforms fibrinogen into fibrin (35). In soft tissue infections, this results in two networks of fibrin, one tight around the bacterial colonies by staphylo-coagulase, which remains close to the bacteria. The other network is wider and is due to von Willebrand factor binding protein being more dispersed (36, 37).

*Staphylococcus aureus* can induce fibrinolysis by secreting staphylokinase, which must form a complex with traces of plasmin present in the medium, thus reducing the plasminogen and producing more plasmin in a kind of vicious circle (38). Via this mechanism depending on quorum sensing, staphylokinase facilitates the bacterial spread and dispersion once the bacterial population becomes too condensed and thus serves as a leak. In IE, staphylokinase may contribute to vegetative degradation and embolization, which may explain why *S. aureus* IEs are more prone to embolization (39).

Recently, a study questioned this classic description of the development of endocardial vegetation. The authors distinguished two models according to the inflammatory or injured state of the valvular endothelium. Thus, they have demonstrated that platelets are more involved in the inflammatory state that corresponds to the above description. In the case of endothelial lesion, it is rather the fibrin network that serves as a link; the platelets have shown a minor presence in this case (40).

### Biofilm Formation

Platelet–*S. aureus* interactions contribute to cell attachment for biofilm formation (41), implied in resistance and adaptation to stress conditions of *S. aureus* (42). Biofilms are a complex mixture of an adhesive matrix composed of extracellular substances that enclose bacteria. In IE, this matrix is deposed on a layer mainly formed of platelets (43). In the case of *S. aureus*, the extracellular matrix is composed of proteins, DNA, and polysaccharide intercellular adhesin (PIA) which is mainly synthesized from UDP-*N*-acetylglucosamine during the exponential growth phase (44).

### Host Response

Deposition of bacteria in the endocardial vegetation triggers an inflammatory process, with recruitment of inflammatory cells. Platelets, as major elements interacting with bacteria, seem to have a contradictory effect. On the one hand, activated platelets surround the bacteria, which protect against host immunity. On the other hand, this platelet coat forces pathogens to form clusters with a lower growth rate than if they were free (45). Platelets also have a secretory activity of an array of antimicrobial compounds grouped as platelet microbicidal proteins and thrombocidins contained in α-granules and platelet β defensin (hBD-1) released under the action of thrombin or staphylococcal α-toxin (45). These proteins showed a significant bactericidal effect on *S. aureus* (45–47).

In addition, platelets interact with *S. aureus* through complement proteins. Indeed, platelets gC1q-R fix bacteria covered by the C1q (24, 48), helping to destroy bacteria. Moreover, P selectin can bind C3b, another complement protein (49, 50).

Beyond the direct lytic actions of platelets on *S. aureus*, it has been shown that platelets are important for the action of other phagocytic cells such as macrophages and neutrophils. Therefore, after activation, platelets are considered as a source of interleukin-1β. This cytokine has been associated with macrophage recruitment and bacterial uptake (51).

Recruited neutrophils to vegetation site, supported by platelets, in which hBD-1 is involved (45), form neutrophil extracellular traps (NETs), a network composed mainly of DNA and histones (52). This extracellular network was found incorporated into the bacterial biofilm inside the vegetation (53). Although NETs prevent the spread of invasive bacteria, they have been shown to contribute to vegetation growth and damage host tissues (54, 55).

## Distinct Effects of Aspirin and Salicylic Acid on *in vitro S. aureus*–Platelet Interaction

Platelet–bacteria interactions have largely been used as an *in vitro* model for studying the pathophysiology of IE or the effect of drugs on it. Regarding aspirin, its effect on interactions between *S. aureus* and platelets has been the subject of numerous *in vitro* studies. Two distinct effects need to be considered: one related to mother molecule, acetyl salicylic acid (ASA), which acts mainly on platelets, and the other, related to salicylic acid (SAL), its main metabolite, which acts on the genetic regulation of the bacterium virulence factors.

### Aspirin Effect

As known, aspirin inhibits cyclooxygenase pathway and reduces platelet activation. Moreover, aspirin significantly decreases the platelet expression of CD62P and CD63 induced by *S. aureus* compared to untreated platelets (56). CD 62P and CD 63 are proteins expressed after degranulation of alpha and dense platelet granules, respectively, and are activation-specific antigens on the platelet surface (57).

Aspirin is linked to the synthesis of triggered lipoxin by aspirin, an analog of lipoxin A4 (58), a metabolite of arachidonic acid, with both anti-inflammatory and antibacterial properties. Lipoxin A4 has been associated with the alteration of bacterial cell membrane, notably on *S. aureus*, contributing to bacterial clearance (59). In addition, it was linked to a decrease in neutrophil recruitment and the formation of neutrophil–platelet aggregates (60). This effect of aspirin via the fatty acid metabolic pathway has been suggested to explain the decrease in mortality in studies of sepsis linked to *S. aureus* (61). However, no studies are currently available on this mechanism triggered by aspirin in IE models.

An infectious environment can trigger platelet apoptosis (62, 63). *S. aureus* has been shown to induce *in vitro* degradation of the Bcl-xL survival protein (64) and an increased expression of markers of cell death (56). Aspirin has been shown to reduce the effect of *S. aureus* on platelet killing, partially preventing thrombocytopenia induced by the bacterium.

Like other nonsteroidal anti-inflammatory drugs, aspirin has antibacterial activity against strains of *S. aureus*. Although individually, with a high minimum inhibitory concentration (MIC), it cannot be used as a standard antibiotic, aspirin has shown a synergistic action with cefuroxime and chloramphenicol by decreasing their own MIC, suggesting its use as an adjuvant in the fight against multidrug-resistant methicillin-resistant *S. aureus*, for which few antibiotics are effective (65).

### Salicylic Acid Effect

SAL modulates the genic expression of several virulence factors of *S. aureus* like α-toxin, adhesins, and biofilm synthesis.

SAL results in the overexpression of sigma factor B operon, which is a two-component regulatory system. This results in the repression of staphylococcal accessory regulator A (Sar A) and accessory gene regulator (Agr), two other two-component regulatory systems in *S. aureus* (66–68).

**Figure 1:**
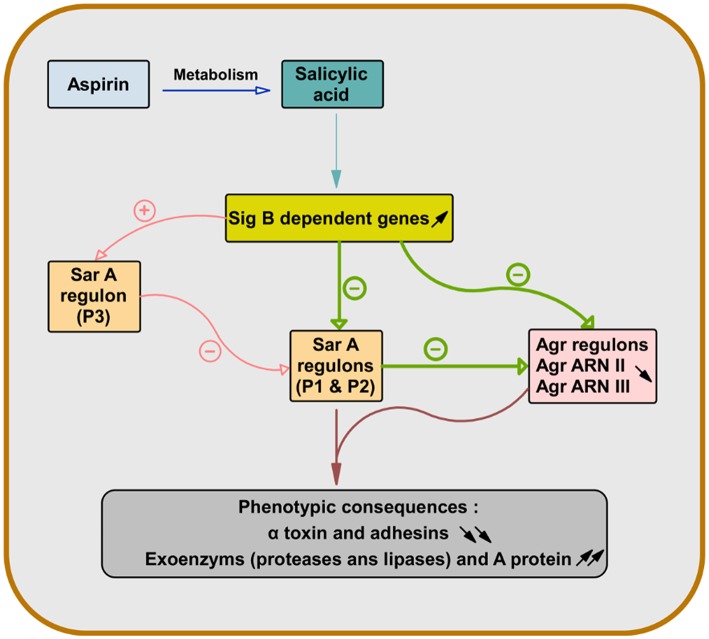
Role of salicylic acid in the gene regulation of *S. aureus* virulence factors. After deacetylation of aspirin, salicylic acid causes overexpression of sigma factor B-dependent genes. This leads to repression of genes dependent on staphylococcal accessory regulator A (SarA) (Sar P1 and P2) and accessory gene regulator (Agr) (Agr RNA II and Agr RNA III) in *S. aureus*. Since the expression of the α-toxin gene (hla) is dependent on both Sar and Agr and the expression of the wall-bound adhesins genes are also controlled by Sar, the pretreatment of *S. aureus* with salicylic acid leads to their attenuation and increases the production of exoenzyme and protein A (66–70).

As the expression of α-toxin (hla) depends on both Sar and Agr and the expression of wall-bound adhesins is also controlled by Sar, pretreatment of *S. aureus* with SAL allowed their attenuation (Figure 1) (69, 70).

Although the α-toxin is associated with lytic action and induction of platelet aggregation, it was linked to the release of platelet antimicrobial proteins. Hyperproduction of α-toxin was associated with a decrease in *S. aureus* virulence through a greater induction of platelet antimicrobial protein release (71, 72). Therefore, the modulation by SAL of the expression of *S. aureus* virulence factors in relation to platelets has contradictory consequences on them. Indeed, although SAL can slow vegetation growth, its repression of α-toxin expression may be responsible for reducing the efficacy of platelet intervention against *S. aureus* in the case of aspirin treatment (70).

**Figure 2:**
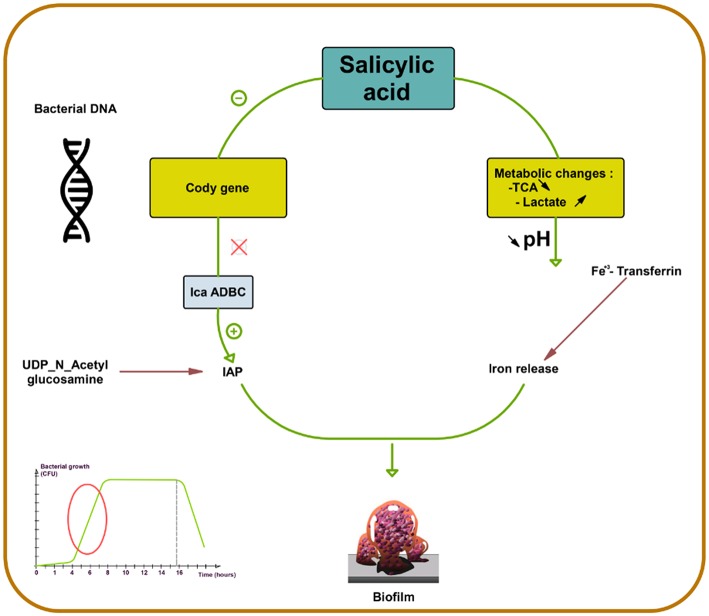
Salicylic acid effect on biofilm formation in *S. aureus*. Salicylic acid (SAL) decreases the activity of tricarboxylic acid (TCA) and increases lactate production by potentiating the lactic fermentation pathway. This makes the medium acidic promoting iron release from human transferrin and its acquisition by *S. aureus*. In addition, SAL reduces the transcription of CodY, a global regulator that controls the expression of metabolism and virulence genes. CodY gene repression by SAL removes repression on icaADBC and thus enhances IAP production from UDP-*N*-acetyl glucosamine. SAL blocks negative control on icaADBC and results in increased biofilm formation (44, 73–75).

SAL strongly favors biofilm production in *S. aureus* by increasing PIA production (73). First, SAL induces a metabolic change, decreasing the external pH, which favors the release of iron from human transferrin and its acquisition by bacteria. Second, SAL increases icaADBC expression in *S. aureus*, the main regulator of PIA synthesis (Figure 2) (44, 74, 75) that contributes to the persistence of infection in patients who are on chronic aspirin therapy (73).

Extracellular adherence protein (Eap) is an adhesin belonging to the group of proteins called the “secreted expanded repertoire adhesive molecules” secreted by *S. aureus*. Eap is mainly expressed during the early stages of infection (76). It acts as a bridge connecting *S. aureus* to host molecules, such as fibrinogen, collagen, and fibronectin (77). It also binds to platelet, triggering their activation (78). Eap is dependent on saeSRS, a two-component *S. aureus* regulatory system (79). SAL has been reported to overexpress saeSRS in *S. aureus* and increases Eap expression, contributing to the persistence of infection (80, 81) (Table 1).

## Animal Experimentation on Aspirin Effect on *S. aureus* Infectious Endocarditis

The benefit of aspirin has been evaluated in several animal studies. However, significant differences must be underlined regarding the concentration, the time of introduction, and the duration of treatment. Indeed, the modulation of these parameters leads to conflicting results.

Kupferwasser et al. demonstrated that the 8 mg/kg/24 h of aspirin dose is the most effective in decreasing vegetation weight and bacterial density. In this study, aspirin was administered for 72 h starting 24 h after inoculation of SAL-precultured *S. aureus* strain. Pretreatment of bacteria by SAL was carried out to simulate the presence of this metabolite within the bloodstream in patients under aspirin therapy. Aspirin resulted in smaller and less friable vegetations, which resulted in a significant reduction in bacterial density in renal lesions and in the degree of renal embolic infarction (82). These results showed the combination of the two distinct effects of ASA and SAL on both platelets and bacteria (65) knowing that, at this dose, aspirin showed no antibacterial effect via the determination of the MIC (83).

**Figure 3:**
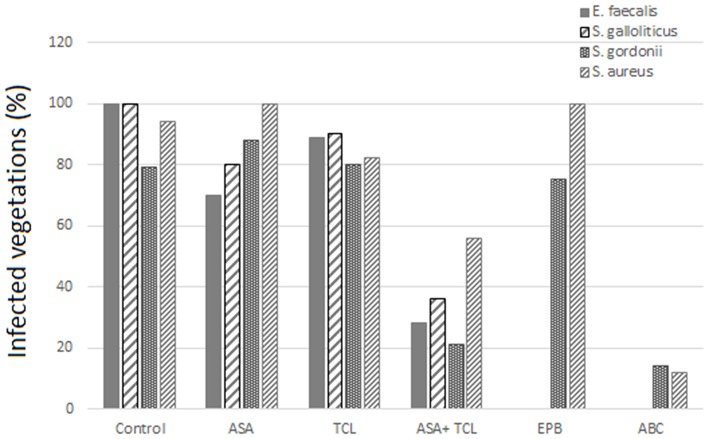
Effect of antiplatelet agents on the rate of infected vegetations in the animal model. Effect of aspirin (ASA), ticlopidine (TCL), alone or in combination, as well as eptifibatide (EPB) and abiciximab (ABC) on the prevention of experimental infective endocarditis induced by *E. faecalis, S. gallolyticus, S. gordonii*, and *S. aureus* “yellow.” Eptifibatide and abciximab in the case of *S. gordonii* and *S. aureus* (84, 86).

**Figure 4:**
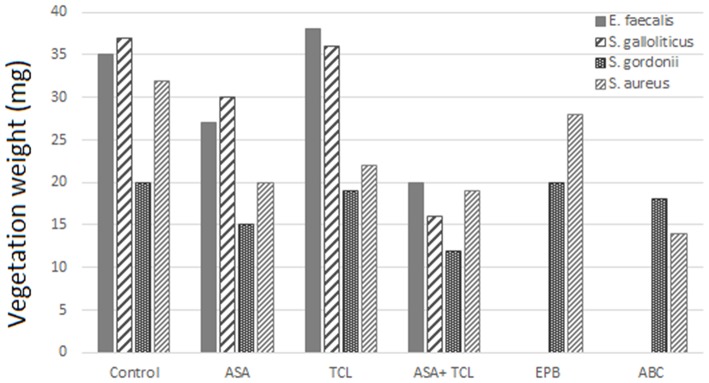
Effect of antiplatelet agents on endocardial vegetation weight in the animal model. Effect of aspirin (ASA), ticlopidine (TCL), alone or in combination, as well as eptifibatide (EPB) and abiciximab (ABC) on weight of vegetation induced by *E. faecalis, S. gallolyticus, S. gordonii*, and *S. aureus*. Eptifibatide and abciximab in the case of *S. gordonii* and *S. aureus* (84, 86).

The combination of aspirin and ticlopidine in a rat model of IE showed higher effect regarding rates of infected vegetations and their weight than aspirin administered alone (84, 85). This combination was subsequently demonstrated to be effective by the same author, not only on *S. aureus* but also on *Streptococcus gordoni, Streptococcus gallolyticus*, and *Enterococcus faecalis* (84, 86) (Figures 3, 4).

According to Veloso et al., the rhythm of bacteria introduction might influence the effect of aspirin. A bolus injection results in a transient bacteremia (1–2 min) of more than 1,000 CFU/ml that can negate the beneficial effect of antiplatelet prophylaxis (84). This model was used by Nicolau et al. and resulted in a nonsignificant decrease, neither in vegetation weight nor in bacterial density (87). Introducing bacteria to have a blood concentration between 2 and 50 CFU/ml, but for a prolonged period, was more realistic and gave a bacteremia comparable to that measured in humans, for example, by skin damage from the injection of impure material. This model of bacterial introduction has been linked to the expression of the beneficial effects of aspirin.

Early combination of aspirin and vancomycin significantly decreased the weight of the vegetation and improved the rate of sterilization compared to vancomycin alone and late ASA associated with vancomycin. These interesting results underlined the benefit of aspirin used in prophylaxis and hypothesized of its use as an adjuvant treatment for antibiotic therapy (83).

A recent experimental study in mice revealed the presence of two distinct vegetation development models, one related to vascular damage *in situ* using fibrin without platelet intervention; the other, on the contrary, involves platelets on an inflammatory valve site. This distinction made by Liesenborghs et al. may suggest when aspirin may be most effective, on the one hand, concerning the involvement or not of the platelets, and on the other hand, concerning the inflammatory status, being aspirin also an anti-inflammatory drug (40).

In addition to the endocarditis model and in relation to the interaction of platelets with other immune cells, an experimental study showed that aspirin significantly decreased NETosis and NET formation, thus reducing the resulting damage (88). These results were confirmed by another experimental study on EI in rats using *Streptococcus mutans* (53). More targeted studies on staphylococcal IE model are therefore needed to confirm or refute these results.

## Clinical Studies

Antiplatelet therapy in clinical studies showed conflicting results. Several studies have hypothesized that the use of antiplatelet drugs may have a beneficial effect on the embolism associated with IE (89–91).

### Embolic Risk

**Figure 5:**
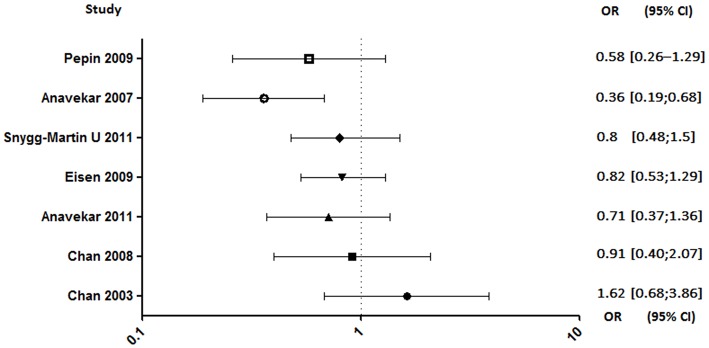
Embolic rate between aspirin and no aspirin users. Odds ratio (OR), 95% confidence interval (95% CI) (4, 89–94).

In seven studies enrolling a total of 2,677 patients, investigating the aspirin effect on embolic risk during IE, four of them concluded that it was not effective (4, 92–94), two showed a benefit in specific cases (90, 91), and only one demonstrated its benefit (Figure 5) (89). Indeed, the only prospective study starting treatment 30 days after the onset of infection was associated with its ineffectiveness in reducing the risk of embolism (4). Same negative results were obtained in a retrospective study conducted by the same author evaluating the effect of long-term aspirin (92). A study distinguishing cases of *S. aureus*-related embolism from other species also concluded that aspirin was ineffective. However, although not significant, the reduction in embolism rate in aspirin-treated patients with staphylococcal endocarditis appeared to be greater compared to other species [*S. aureus*: adjusted odds ratio (AOR), 0.44; 95% confidence interval (CI), 0.13–1.50, *p* = 0.19; other pathogens: AOR, 0.70; 95% CI, 0.23–2.13; *p* = 0.53; all: AOR, 0.58; 95% CI, 0.26–1.29; *p* = 0.18] (94). Another study, which focused only on staphylococcal IE, reported that aspirin was associated with a significant negative univariate analysis with all emboli. Despite a loss of this significance with multivariate analysis, aspirin remained a significant predictor of decreased risk of acute valvular surgery (*p* < 0.04) without increased bleeding stroke (90).

In a larger cohort, Anavekar et al. demonstrated that a daily dose of aspirin at 81 mg/day before the onset of IE was associated with a significant decrease in the embolic event, mainly cerebral ones (89). However, the author did not confirm their results in a second study, in which the effect of antiplatelet therapy on embolic risk was not statistically significant (93). This discrepancy was explained by a critical difference between the two cohorts on the frequency of cardiovascular risk factors for which antiplatelet agents have been prescribed.

In another study, it has been reported that there was no effect of antiplatelet therapy on cerebrovascular complications. However, among episodes related to *S. aureus*, there was a tendency to have fewer cerebrovascular complications in patients with established antiplatelet therapy, suggesting that there may be a beneficial effect of antiplatelet therapy in the case of staphylococcal IE (91).

### Mortality

**Figure 6:**
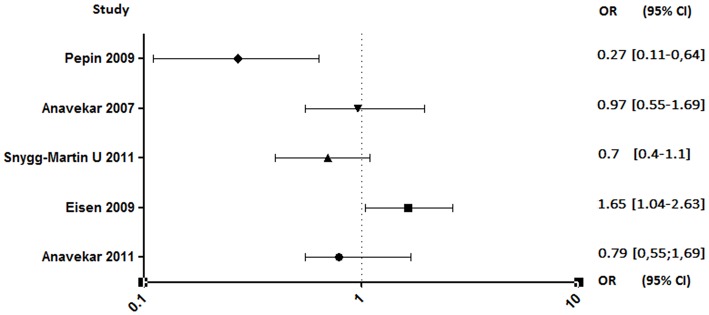
Mortality rate between aspirin and no aspirin users. Odds ratio (OR), 95% confidence interval (95% CI) (89–91, 93, 94).

Both studies conducted by Anavekar et al. were related to aspirin ineffectiveness on 6 months mortality (89, 93). Moreover, Eisen et al. reported that overall 1-year mortality among aspirin users was significantly higher (90). In contrast, the study conducted by Pepin et al. has shown that mortality related to IE, all species combined, decreased significantly in patients on chronic antiplatelet therapy (AOR, 0.27; 95% CI, 0.11–0.64), although this decrease is not significant when taking only *S. aureus* IE (AOR, 0.46; 95% CI, 0.14–1.55) (Figure 6) (94).

### Hemorrhagic Risk

**Figure 7:**
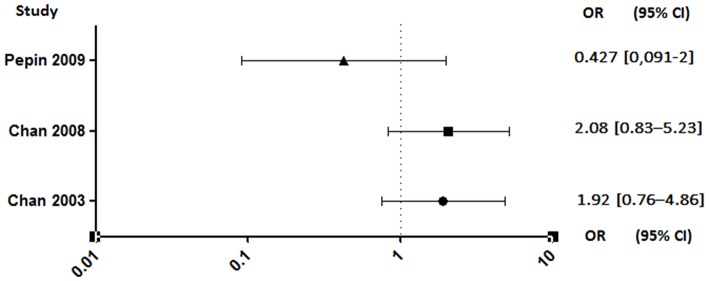
Hemorrhagic risk between aspirin and no aspirin users. Odds ratio (OR), 95% confidence interval (95% CI) (4, 92, 94).

Unlike anticoagulants, which have shown an extremely high risk of bleeding in animals (95–97) or even in clinical studies interrupted soon after a high rate of cerebral hemorrhage (98), antiplatelet agents appear to have a lesser effect. Despite this, a rigorous benefit/risk ratio assessment should be established for the use of aspirin in patients who are already at high risk of bleeding (99). The results are also discordant regarding this parameter. Indeed, studies conducted by Pepin et al. and Anavekar et al. showed no increase in bleeding risk in patients on aspirin (89, 94). By opposite, the two studies conducted by Chan *et al*. showed that antiplatelet therapy was associated with an increased risk of hemorrhage, although not significant (Figure 7) (4, 92).

Finally, a meta-analysis performed on nine clinical studies involving a total of 5,400 patients, in which 1,230 were treated with aspirin, demonstrated that aspirin is associated with a significant decrease in major systemic embolism, both with chronic and acute use [odds ratio (OR), 0.66; 95% CI, 0.54–0.81], and that the risk of bleeding tends to decrease (OR, 0.71; 95% CI, 0.44–1.14); however, it will be linked to an increased risk of death without being significant (OR, 1.20; 95% CI, 0.97–1.50) (100).

### Limitations

Overall, we must be careful about the interpretation of the results of these clinical studies. First, in addition to the small sample sizes in most studies, which may be insufficient to reveal significant differences, the composition of the two populations under and without antiplatelet therapy was heterogeneous in each study with a higher mean age in treated patients. This gives a high Charlson index and a higher comorbidity, which is an extremely influential confounding factor of mortality. Second, the timing of initiation of antiplatelet therapy before or after diagnosis of IE showed a difference in the rate of embolism, suggesting that this treatment has a much more prophylactic effect. However, although a rat model of staphylococcal EI demonstrated the benefit of the combination of aspirin and ticlopidine in preventing the formation of vegetation, we have no data yet confirming the benefit of aspirin in prophylaxis in humans (101). Third, the dose of daily aspirin also appears to have an impact on the results with a more expressed effect when administered at low doses. Indeed, in the Anavekar study, most patients received low-dose aspirin therapy (?81 mg/day) (89) compared to the 325 mg/day dose in the two studies of Chan et al. (4, 92). Unlike lower doses of aspirin, higher doses can inhibit prostacyclin production mainly by endothelial cells with an antiplatelet effect and thus cause an adverse effect (82). The sample size must also be taken into consideration. For example, in Chan's study, only 31% of the target sample size was recruited. The test may not be enough to detect a slight beneficial effect of aspirin on the risk of embolism. In addition, in all these studies, no evaluation of patient's sensitivity to antiplatelet agents was reported, even though these molecules show a significant resistance rate (102, 103).

Finally, the embolism rate and the impact of the antiplatelet agents must take into consideration the infectious species involved, since the thrombus formation process depends on it (90). This condition has not been addressed in most of these studies. Mixed results may mask a beneficial effect on a specific bacterial species.

## Conclusions

The use of aspirin was first proposed as a preventive treatment for all forms of IE. Older studies have been performed to determine the appropriate dose, the delay in preventive or curative treatment, and the various combinations with other antiplatelet drugs. Despite these efforts, the benefit of aspirin in this indication remains unclear. New *in vitro* and experimental studies indicate that the development of endocardial vegetation occurs differentially depending on the initial valvular state of the patient and the implicated bacterium. Today, more targeted approaches have already begun concerning the selection of patients eligible for this type of treatment according to these two primordial parameters, namely, the type of lesion and the bacterial strain involved.

## Author Contributions

NH, LC-J, and GH have made a substantial, direct, and intellectual contribution to the work and wrote and approved it for publication.

### Conflict of Interest

The authors declare that the research was conducted in the absence of any commercial or financial relationships that could be construed as a potential conflict of interest.
